# Microlens-Assisted Light-Scattering Imaging of Plasmonic Nanoparticles at the Single Particle Level

**DOI:** 10.3390/bios13090871

**Published:** 2023-09-06

**Authors:** Pengcheng Zhang, Tingting Zhan, Sha Xue, Hui Yang

**Affiliations:** Bionic and Intelligence Sensing Center, Institute of Biomedical and Health Engineering, Shenzhen Institutes of Advanced Technology, Chinese Academy of Sciences, Shenzhen 518052, China; pc.zhang@siat.ac.cn (P.Z.); tt.zhan@siat.ac.cn (T.Z.);

**Keywords:** plasmonic nanoparticle, dielectric microsphere, microlens, light-scattering imaging, single-nanoparticle imaging, high temporal resolution

## Abstract

We present a microlens-assisted imaging approach to record the scattering light of plasmonic nanoparticles at the single particle level. The microlens can significantly enhance the backscattering of visible light from individual plasmonic nanoparticles by several dozen folds, and single gold nanoparticles with a diameter as low as 60 nm can be imaged under a conventional optical microscope. This can benefit from a significant increase in the scattering intensity afforded by the microlens, meaning that the imaging of gold nanoparticles at a high temporal resolution (up to 5000 Hz) can be achieved, which is fast enough to record single particle adhesion events on the substrate. This research presents a fast and efficient means of acquiring scattering light from plasmonic nanoparticles, which has great potential to develop plasmonic nanoparticle-based biosensors and investigate a wide range of plasmonic nanoparticle-based fast interaction processes.

## 1. Introduction

Metallic nanoparticles support the collective oscillation of conduction band electrons induced by incident electromagnetic radiation, which is known as the phenomenon of localized surface plasmon resonance (LSPR). LSPR induces the strong enhancement of local electromagnetic fields and is highly sensitive to the environment surrounding metallic nanoparticles. The acquisition and analysis of LSPR spectral information has, thus, been widely used in many research directions, including surface-enhanced Raman spectroscopy (SERS), biosensing, surface catalysis, plasmonic tags, particle tracing, and many others [1,2,3,4,5,6,7,8]. These metallic nanoparticles are also termed plasmonic nanoparticles and exhibit unique and superior light scattering at resonant wavelengths. However, the scattering light that carries the LSPR spectral information of single plasmonic nanoparticles is relatively weak and decreases dramatically with size [9,10]. Driven by novel approaches to illumination, detection, and background suppression, recent advances in scattering-based microscopy have reached remarkable sensitivity. Notably, these advances encompass techniques, such as dark-field imaging, interferometric detection, and surface plasmon resonance microscopy. However, these techniques usually require a long acquisition time, high-power illumination sources, and special objectives to isolate and maximize the smallest possible scattering signals. This results in limited temporal resolution and expensive instrument costs, which pose significant challenges in terms of their accessibility and widespread adoption [11,12,13,14]. In addition, these limitations have hindered their application as practical tools for various purposes such as sensing and diagnostics. There is a strong demand for efficient and simplified methods to image and collect scattering light from single plasmonic nanoparticles.

Recent studies have shown that dielectric microspheres with an appropriate refractive index can be utilized as microsphere lenses (microlens) to provide an image resolution beyond the diffraction limit [15,16,17,18,19]. In addition, these microlenses can converge the incident light, which could enhance the backscattering light from nanoparticles located within its field of view [20,21,22]. These properties give microlenses intrinsic advantages to collect the faint scattering light generated by individual nanoparticles, as well as to detect and manipulate them [23,24,25,26,27,28]. Employing microlenses for plasmonic nanoparticle imaging offers a straightforward optical configuration and is anticipated to amplify the intensity of their scattered signal. Here, in this study, we report that dielectric microspheres can be utilized as microlenses to achieve fast and efficient light-scattering imaging of single plasmonic nanoparticles under a conventional light microscope. We describe the imaging principle and demonstrate the imaging of gold nanoparticles as small as 60 nm. In addition, we employ this technique to record and analyze individual particle adhesion events on the substrate, thereby showcasing its exceptional temporal resolution up to 5000 Hz.

## 2. Materials and Methods

### 2.1. Materials

The powder of high refraction index glass microspheres (~22 µm, refractive index 1.92) was purchased from Microspheres-Nanospheres (Cold Spring, NY, USA). 3-Aminopropyl triethoxysilane (APTES) was purchased from Aladdin Bio-Chem Technology (Shanghai, China). Gold nanoparticles (AuNPs, citrate stabilized) were purchased from BBI Solutions (Crumlin, UK). Polydimethylsiloxane (PDMS) was obtained from Dow Corning (Midland, MI, USA). Milli-Q water was used in all the experiments.

### 2.2. Methods

#### 2.2.1. Preparation of the Microlenses

To fix and stabilize the microlenses in a solution, glass microspheres were embedded in the cured PDMS layer on the glass substrate. Briefly, the glass substrate (thickness 1 mm) was cleaned with ethanol, followed by rinsing with deionized water and drying with nitrogen. Subsequently, a suspension containing glass microspheres (dissolved in water) was deposited onto the substrate and then dried at 70 °C for 15 min. Once the suspension was fully dried, a layer of PDMS with a mixing ratio of 10:1 was spin-coated onto the glass substrate. The thickness of the PDMS layer was carefully adjusted by tuning the coating speed and coating time to ensure that the top surface of the microspheres was exposed. In our experiments, spin coating was performed at 5000 rpm for 5 min. After spin coating, the microlens chip was subjected to a de-bubbling process and cured at 70 °C for 1 h to solidify the PDMS layer. After the PDMS layer was cured, the chip was treated with air plasma (30 W, 2 min). Subsequently, it was immersed in a 5% APTES solution at a temperature of 60 °C in a water bath for 30 min. These treatments induced positive surface charges on the chip, facilitating an electrostatic attraction to the negatively charged AuNPs present in the solution.

#### 2.2.2. Microlens Assisted Imaging of the Plasmonic Nanoparticles

**Figure 1:**
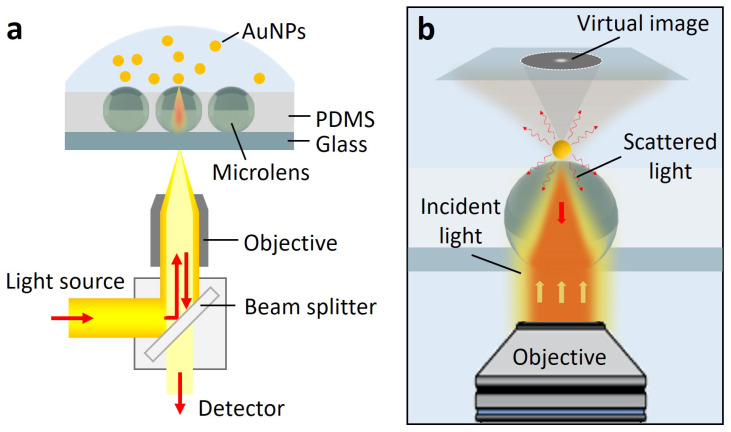
(**a**) Schematic illustration of the optical configuration. The observation was conducted through an inverted microscope under a reflected optical path. The microlenses were embedded in the PDMS layer and positioned above the objective. The AuNP solution was dropped on the microlenses. (**b**) Illustration of the formation of the virtual image using the microlens. The microlens act as an auxiliary lens that forms a magnified virtual image above the specimen’s surface. Upon illumination, the scattered light of AuNPs is collected by the microlens and finally recorded and imaged by the optical microscope.

To conduct imaging experiments, AuNPs were used as a representative sample. In total, 30 μL of the AuNPs solution was applied onto the microlens chip, followed by an observation with an inverted optical microscope equipped with a white light source (white LED light source, ZEISS Colibri, ZEISS, Oberkochen, Germany), as depicted in Figure 1. A 40× objective (ZEISS LD A-Plan) with a numerical aperture (NA) of 0.55 was used. The objective was focused on the imaging plane of the microlens. The backscattering signal of AuNPs was collected by the objective and finally transmitted to a sCMOS camera (Hamamatsu C13440, Hamamatsu, Shizuoka, Japan). The exposure time was set between 166 μs and 20 ms, and the region of interest (ROI) was adjusted accordingly. The intensity of the AuNP is represented by the grayscale values measured from the images. Only the AuNPs located in the ROI were included in the statistics.

#### 2.2.3. Simulation of Photonic Hotspot Generated by the Microlens

A simulation based on the finite element method (FEM) was conducted to study the generation of the photonic hotspot by microlenses. The microlens was assigned a refractive index of 1.92, while the surrounding medium was set at 1.43 for the PDMS layer and 1.33 for the water solution. A monochromatic plane wave with a wavelength of 600 nm was set to propagate from the bottom of the microlens. The color map in the simulation corresponded to the magnitude of the electric field, as represented by |E|^2^ (V/m).

## 3. Results and Discussion

The optical configuration is based on a conventional inverted optical microscope equipped with a low NA objective lens (40×, NA = 0.55) and illuminated under an unpolarized incoherent white light source, as illustrated in Figure 1a. The microlenses were embedded in the PDMS layer and positioned above the objective (details can be found in the Methods section). The AuNP solution was dropped on the microlenses. The surface of the microlens possesses positive charges, which produces an electrostatic attraction to the negatively charged AuNPs in the solution. The AuNPs can be attracted to the surface of the microlens. The microlens act as an individual auxiliary lens that can generate a virtual image above the specimen’s surface. Here, in the reflection-based illumination mode, the backscattered light of AuNPs can be collected by the microlenses, captured by the objective, and finally imaged onto the detector, generating the magnified virtual image of AuNPs in the far field (Figure 1b).

**Figure 2:**
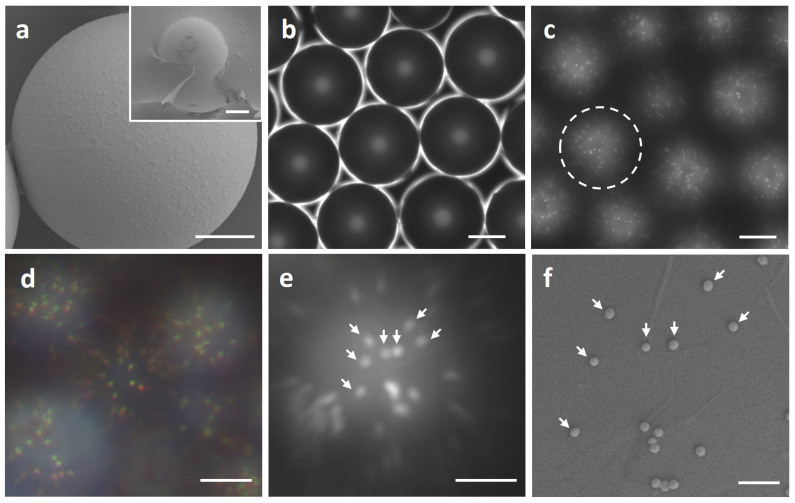
(**a**) Scanning electron microscope (SEM) image of the individual microlens. The inset image shows the embedded microlens. The top surface of the microlens is exposed out of the PDMS layer. Scale bar = 5 µm. (**b**) The optical image of the microlens array in the solution under the inverted microscope with a reflected optical path. The focus is on the contour of microlenses. Scale bar = 10 µm. (**c**) Gray level virtual image of 100 nm AuNPs captured under a microlens. The dashed circle indicates the position of a microsphere lens. The bright spots in the image indicate the individual AuNPs. The focus is on the virtual image plane of the microlens. Scale bar = 10 µm. (**d**) Colored image (captured by a CCD camera) of 100 nm AuNPs under a microlens with white light illumination. Scale bar = 10 µm. The color exhibited by the AuNPs was caused by the surface plasmon resonance phenomenon. AuNPs imaged under a microsphere lens (image (**e**)) and corresponding AuNPs imaged with SEM (image (**f**)). The white arrows indicate their positions for comparison. Scale bar = 5 µm in image e and scale bar = 500 nm in image (**f**).

The morphology of the microlens is characterized by the scanning electron microscope (SEM), as shown in Figure 2a. The microlenses are embedded within the PDMS layer, with their top surfaces exposed, as shown in the inset image of Figure 2a. This ensures that objects positioned above the microlens remain within the focal range, thereby enabling the microlens to effectively focus. Microlenses are relatively uniformity in size and are closely packed, forming a microlens array, as shown in Figure 2b. This microlens array enables massive parallel imaging. In the presence of AuNPs in the solution, AuNPs adhere to the surface of the microlens due to an electrostatic force. The backscattered light of the AuNPs is collected and imaged by the microlenses. Under the ×40 objective lens, each microlens generates a virtual image where a single AuNP appears as a bright spot-like point-spread-function (PSF) against the background in the field-of-view (FOV) of each microlens, as shown in Figure 2c. Single AuNP can be distinguished clearly with a high signal-to-noise ratio in the image. The high-quality imaging area is located at the geometric center of the microlens. Toward the contour of the microlens, the quality of the imaging, as well as the brightness of AuNPs, decreases, which is a typical feature in microlens-assisted imaging. The colored image in Figure 2d shows that AuNPs exhibit a yellowish color under white light illumination, which is caused by the surface plasmon resonance phenomenon. The virtual image of AuNPs through the microlens was also compared with SEM images, as shown in Figure 2e,f. The AuNPs in the microlens image and SEM image, marked by white arrows, correspond to the same individual AuNPs. Aggregated AuNPs can be distinguished by their size. The FOV of the microlens, which is defined as the diameter of the circle at the sample surface where the round shape of AuNPs is able to be discerned, was determined to be around 3.2 µm for the 22 µm microlens, which is consistent with the reported results [29].

**Figure 3:**
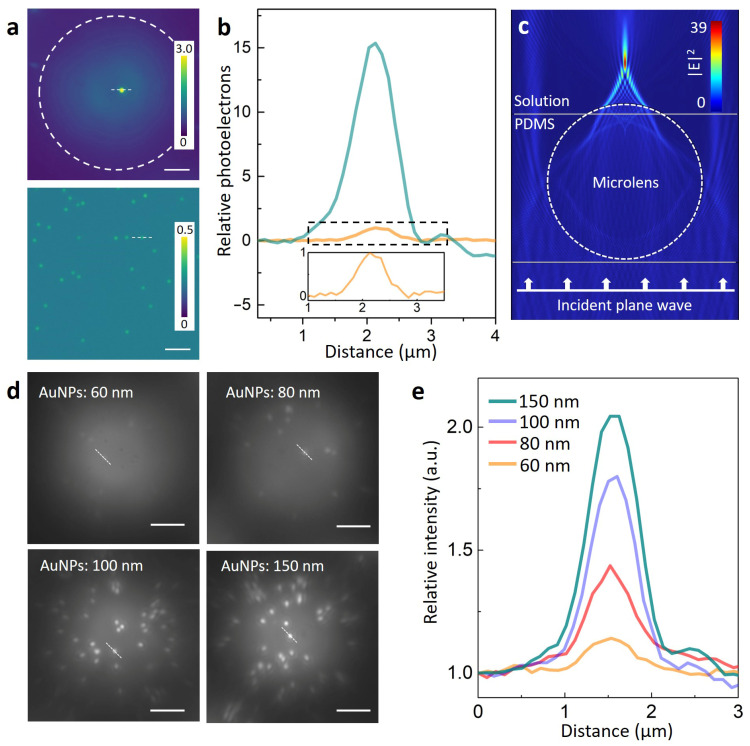
(**a**) Photoelectron images of 150 nm diameter AuNPs imaged with (top image) and without (bottom image) the microlens. Photoelectron scale = photoelectrons/10^4^. The images were acquired at an exposure time of 10 ms with white light illumination (power intensity = 78 mW/cm^2^). Scale bar = 5 µm. Note that for the convenience of visual identification, the scale bars of the photoelectrons are different. (**b**) Relative photoelectrons from individual AuNPs indicated by dashed lines in image a. The green and orange lines denote the AuNPs imaged with and without the microlens, respectively. Their photoelectrons were normalized by the maximum value of AuNPs imaged without the microlens. Insert: the enlarged view of AuNPs imaged without the microlens. (**c**), Finite element method simulation of the electric field (|E|^2^) distribution generated by the microlens. The diameter of the microlens was set at 22 μm, and the wavelength of the illumination light was set at 600 nm. The simulation shows that the incident light was converged by the microlens at its shadow side. (**d**) Gray-level images of AuNPs of different diameters (150 nm, 100 nm, 80 nm, and 60 nm, respectively) imaged under a microlens. Images were acquired at an exposure time of 1 ms with white light illumination (power intensity = 1 W/cm^2^). (**e**), Cross-section profile of the corresponding AuNPs (indicated by the white dashed line) from image (**d**).

These microlenses can converge the incident light and enhance backscattering light from the AuNPs located within its FOV. These properties give microlenses intrinsic advantages to collect faint scattered photons from individual AuNPs. As shown in Figure 3a, we compared the scattering intensity of 150 nm diameter AuNPs imaged with and without the use of microlenses. The grayscale images captured by the sCMOS camera were converted and displayed in terms of photoelectrons with the aim of comparing their photoelectron counts. As can be seen clearly from Figure 3a, AuNPs imaged with a microlens exhibited bright visible intensity, while for those imaged without the microlens, only an inconspicuous intensity was observed. It can be noted that for the convenience of visual identification, the scale bars of the photoelectrons were different. The integration of net photoelectrons originating from individual 150 nm AuNPs indicates that the microlens significantly enhanced the detectable photon count by approximately 40 times under the same level of illumination power (Figure 3b). A simulation based on the finite element method (FEM) further confirmed the optical focusing property of the microlens (Figure 3c). The simulation results showed that the incident light could be converged at the shadow side of the microlens. Therefore, high-intensity converging light can illuminate plasmonic AuNPs and enhance the backscattering light excited from individual AuNPs. Enhanced backscattering light could also be efficiently collected by the microlens in the far field, which is known as the enhanced backscattering effect [20]. The significant increase in photon abundance enabled us to image single AuNPs with a millisecond temporal resolution under low-power illumination conditions. As shown in Figure 3d, AuNPs of different sizes (60 nm, 80 nm, 100 nm, and 150 nm, respectively) were imaged at an exposure time of 1 millisecond with white light illumination. AuNPs with a diameter of 60 nm only exhibited glimmer bright spots in the image, while larger AuNPs exhibited evident bright spots. As expected, the brightness of the AuNPs was shown to increase with their size (Figure 3e), which originated from their size-dependent scattering cross-section [30].

**Figure 4:**
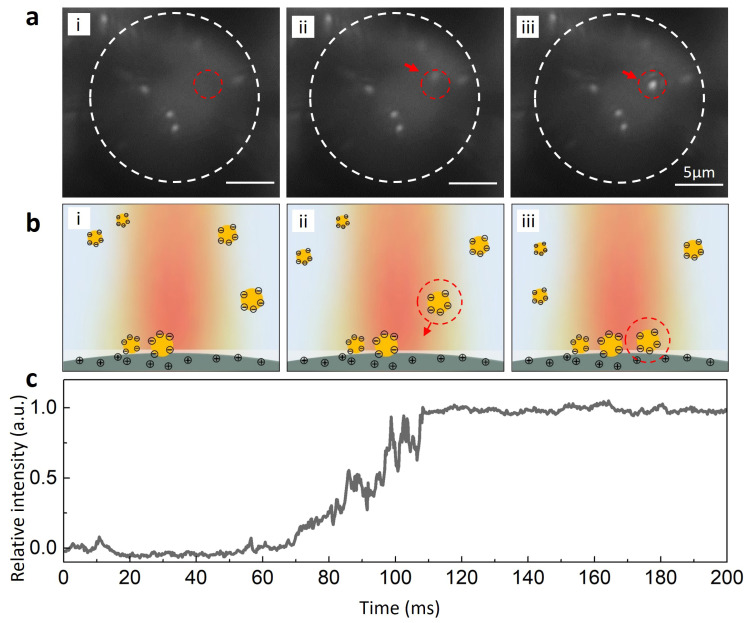
Single particle adhesion event monitored by the microlens. (**a**) Real-time images of an adhesion event at the single particle level from a video recorded at 5000 Hz (corresponding to an exposure time of 200 μs). Video was acquired at an exposure time of 200 μs with white light illumination. The red dashed circle indicates the area where a single AuNP (100 nm) appeared (image ii) and became resident on the microlens surface (image iii). (**b**, i–iii) Schematic illustration of the corresponding adhesion event. (**c**) Intensity of the adhesion area (as indicated in image (**a**) with a red dashed circle (i)) recorded at 5000 Hz under the microlens. The relative intensity value represents the normalized mean intensity of the adhesion area. At time ~70 ms, the AuNP appeared, and the intensity started to increase. At time ~110 ms, the AuNP adhered, became resident on the microlens surface, and the intensity was maintained as stable.

Benefiting from the enhanced backscattering effect, more scattering light could be collected and transmitted to the detector, thus allowing for the imaging and recording of AuNPs at a shorter exposure time and high temporal solution. As a demonstration, we monitored the single particle adhesion event with the microlens. Due to the electrostatic force, AuNPs in the solution could adhere to the microlens surface due to the electrostatic force. This process typically occurs within the timescale of tens to hundreds of milliseconds, depending on the diffusion property of the nanoparticles and the strength of force interactions in the microenvironment. With the help of the microlens, the single particle adhesion event was clearly recorded at a high temporal resolution. As shown in Figure 4a,b, the process of the adhesion event could be recorded at a single particle level up to 5000 Hz (equivalent to an exposure time of 200 μs). This temporal resolution aligns with the capabilities of cutting-edge imaging techniques operating within such short timescales. However, this achievement stands out for its utilization of a significantly simplified optical configuration within a conventional optical microscopy system. In addition, this accomplishment was made possible with an illumination power of below 1 W/cm^2^, which is over a hundredfold lower than the typically required value. The process of single AuNP emergence and settling could, thus, be clearly observed. In particular, the intermediate state of this process, which is shown in image ii in Figure 4a, indicates the origin of the settling AuNP. The intensity traces from the adhesion area show that AuNPs appeared and were resident on the microlens at a ~40 ms time scale (Figure 4c). The ability to image and record nanoparticles at the single particle level with a high temporal resolution provides a platform for investigating a wide range of plasmonic nanoparticle-based fast interaction processes. Although the single FOV for each microlens was limited, further developments could utilize microlenses with a larger size and appropriate refractive index that offer an improved FOV and enhancement capability, aiming to meet the demands of other applications, such as biophotonics and diagnostics. Furthermore, this technique has the potential to be integrated with single-particle spectroscopy technology to measure scattering spectra. This integration could lead to the development of biosensors based on single plasmonic nanoparticles.

## 4. Conclusions

In conclusion, we demonstrated the microlens-assisted light-scattering imaging of plasmonic nanoparticles. This imaging approach efficiently collects the enhanced backscattering light from plasmonic nanoparticles under a conventional optical microscope. Gold nanoparticles down to 60 nm can be imaged and well distinguished at the single particle level. This microlens-assisted imaging approach is able to image individual plasmonic nanoparticles at a temporal solution up to 5000 Hz. Monitoring individual nanoparticle adhesion events on the substrate was achieved on a millisecond scale. The reported results have the potential for developing plasmonic nanoparticle-based biosensors and investigating a wide range of plasmonic nanoparticle-based fast interaction processes.

## Data Availability

Original and raw data files are available from the authors upon reasonable request.
